# Impaired 17,20-Lyase Activity in Male Mice Lacking Cytochrome *b*_5_ in Leydig Cells

**DOI:** 10.1210/me.2015-1282

**Published:** 2016-03-14

**Authors:** Varun Sondhi, Bryn M. Owen, Jiayan Liu, Robert Chomic, Steven A. Kliewer, Beverly A. Hughes, Wiebke Arlt, David J. Mangelsdorf, Richard J. Auchus

**Affiliations:** Departments of Pharmacology (V.S., B.M.O., S.A.K., D.J.M.) and Molecular Biology (S.A.K.) and the Howard Hughes Medical Institute (D.J.M.), University of Texas Southwestern Medical Center, Dallas, Texas 75390; Departments of Internal Medicine and Pharmacology (J.L., R.J.A.) and the Michigan Metabolomics and Obesity Center (R.C.), University of Michigan, Ann Arbor, Michigan 48109; and the Institute of Metabolism and Systems Research (B.A.H., W.A.), University of Birmingham, Birmingham B15 2TT, United Kingdom

## Abstract

Androgen and estrogen biosynthesis in mammals requires the 17,20-lyase activity of cytochrome P450 17A1 (steroid 17-hydroxylase/17,20-lyase). Maximal 17,20-lyase activity in vitro requires the presence of cytochrome *b*_5_ (b5), and rare cases of b5 deficiency in human beings causes isolated 17,20-lyase deficiency. To study the consequences of conditional b5 removal from testicular Leydig cells in an animal model, we generated *Cyb5*^flox/flox^:Sf1-*Cre* (LeyKO) mice. The LeyKO male mice had normal body weights, testis and sex organ weights, and fertility compared with littermates. Basal serum and urine steroid profiles of LeyKO males were not significantly different than littermates. In contrast, marked 17-hydroxyprogesterone accumulation (100-fold basal) and reduced testosterone synthesis (27% of littermates) were observed after human chorionic gonadotropin stimulation in LeyKO animals. Testis homogenates from LeyKO mice showed reduced 17,20-lyase activity and a 3-fold increased 17-hydroxylase to 17,20-lyase activity ratio, which were restored to normal upon addition of recombinant b5. We conclude that Leydig cell b5 is required for maximal androgen synthesis and to prevent 17-hydroxyprogesterone accumulation in the mouse testis; however, the b5-independent 17,20-lyase activity of mouse steroid 17-hydroxylase/17,20-lyase is sufficient for normal male genital development and fertility. LeyKO male mice are a good model for the biochemistry but not the physiology of isolated 17,20-lyase deficiency in human beings.

Androgen biosynthesis requires the enzyme steroid 17-hydroxylase/17,20-lyase (P450 17A1) to convert 21-carbon steroids to 19-carbon steroids. P450 17A1 from all species studied catalyzes 17-hydroxylation with similar rates for substrates pregnenolone and progesterone (Prog), yielding 17-hydroxypregnenolone and 17-hydroxyprogesterone (17OHP), respectively. The 17,20-lyase reaction, however, shows species-specific substrate preferences, with human P450 17A1 catalyzing the conversion of 17-hydroxypregnenolone to dehydroepiandrosterone about 50-fold more efficiently than 17OHP to androstenedione (AD), whereas the rodent enzymes use both pathways. The 17,20-lyase reaction is more vulnerable than the 17-hydroxylase reaction to disruption from the abundance of its electron transfer protein cytochrome P450-oxidoreductase (POR) (1), to mutations in POR, and to phospholipid composition. Because the 17,20-lyase activity is the sole gateway to all androgens, the potent P450 17A1 inhibitor abiraterone was developed for the treatment of castration-resistant prostate cancer (2, 3). The prodrug abiraterone acetate suppresses testosterone (T) synthesis in prostate cancer patients and normalizes androgen production in adult women with uncontrolled classic 21-hydroxylase deficiency (4).

Cytochrome *b*_5_ (b5) is a small, highly conserved 15-kDa hemoprotein cofactor for multiple oxidative reactions, including the metabolism of fats and steroids, reduction of methemoglobin to hemoglobin, and the catabolism of xenobiotics and drugs (5–9). The full-length microsomal form of b5 is found on the cytoplasmic side of the endoplasmic reticulum in many tissues, and a soluble form lacking the C-terminal membrane anchor is abundant in erythrocytes (10). Multiple studies since the 1960s have confirmed the importance of b5 as a modifier of various cytochrome P450 activities, although its mechanism of action remains controversial and might vary with P450 isoforms and substrates. Several mechanisms have been proposed, including direct transfer of electrons to P450s from either reduced nicotinamide adenine dinucleotide-b5 reductase or POR (11) vs an allosteric effect on the P450s and/or POR (12, 13). Depending on the substrate and enzyme, b5 can serve as an obligate component of the reaction or as a modifier of a reaction for the same P450. For example, P450 2B4-catalyzed metabolism of methoxyflurane shows an absolute requirement for b5 (14); in contrast, b5 inhibits the metabolism of benzphetamine by P450 2B4 (15). In the P450 4A subfamily, which consists of eicosanoid and fatty acid hydroxylases, b5 can increase or decrease the Michaelis constant of the reaction, depending on the substrate involved (16).

P450 17A1 also shows substrate-specific modulation via b5 (12). Similar to the substrate-specific modulation of activity observed with b5 and other P450s, b5 stimulates the 17, 20-lyase reaction rate more than 10-fold when 17-hydroxypregnenolone or 17OHP are substrates, but b5 only increases the conversion of 5α-pregna-3α,17α-diol-20-one to androsterone, the most efficient 17,20-lyase reaction for human P450 17A1, by 3-fold (17, 18). Evidence from experiments with apo-b5, which lacks the heme and thus cannot transfer electrons, suggests that b5 action on P450 17A1 does not involve direct electron transfer (12), and b5 might act as an allosteric modulator, promoting interaction of POR with P450 17A1 and/or conformational changes that enhance 17,20-lyase activity.

Combined 17α-hydroxylase/17,20-lyase deficiency is found in rare patients, and isolated 17,20-lyase deficiency is one of the rarest hereditary defects in steroidogenesis (19). Genetic screening allowed the identification of mutations in both P450 17A1 and POR that almost completely ablate 17,20-lyase activity yet only partially impair 17-hydroxylase activity (20). Most recently, 2 reports have identified consanguineous families with isolated 17,20-lyase deficiency due to mutation in the *CYB5A* gene encoding b5 (21). Unlike other forms of isolated 17,20-lyase deficiency, patients with b5 defects demonstrate complete preservation of 17-hydroxylase activity, confirming the in vivo importance of b5 in stimulating the 17,20-lyase reaction.

The in vivo functions of b5 have recently been explored with mouse models of b5 deletion, including a hepatic only deletion (22) and a complete b5-null mouse (23). Complete deletion of b5 causes profound changes in the metabolism of various substrates, in accordance with its role as a regulator of numerous P450 activities. Surprisingly, McLaughlin et al (23) observed only a 50% reduction in intratesticular T in b5-null animals, despite a profound defect in conversion of 17OHP to AD. The b5-null mice, unlike human patients with b5 mutations, were fertile and anatomically indistinguishable from wild-type (WT) littermates (23). Nevertheless, the contribution of altered hepatic T metabolism, which is also a b5-dependent process, confounds the interpretation of these studies. To further explore the importance of b5 in the P450 17A1-catalzyed 17,20-lyase reaction, we have generated a mouse model with deletion of b5 in testicular Leydig cells but not the liver. Through in vivo analysis of androgen synthesis and in vitro analysis of P450 17A1 activity, we demonstrate the physiologic importance of b5 activating the 17,20-lyase reaction and the production of 19-carbon sex steroids from 21-carbon precursors.

## Materials and Methods

### Generation of animals, breeding experiments, and histology

The *Cyb5*^flox/−^ mice were generously provided by Professor C. Roland Wolf and Dr Colin J. Henderson, Cancer Research UK, Ninewells Hospital and Medical School, Dundee, United Kingdom via Dr Hao Zhu, University of Kansas. *Cyb5*^flox/flox^ mice were maintained by random breeding on a 129P2 * C57BL6 genetic background, and Sf1-*Cre* transgenic mice were maintained on a C57BL6 background as described (24). *Cyb5*^flox/flox^:Sf1-*Cre* mice were backcrossed to *Cyb5*^flox/flox^ mice to generate conditional b5 knockout animals. The presence of the floxed *Cyb5* alleles and Sf1-*Cre* transgenes was determined as previously described (22, 25). *Cyb5*^flox/flox^:Sf1-*Cre*:*Srd5a1*^−/−^ mice were generated by crossing *Cyb5*^flox/flox^:Sf1-*Cre* with *Srd5a1*^−/−^ mice. Mice were genotyped for the presence of Srd5a1 as previously described (26). Male mice were studied to provide interpretable data focusing on androgen synthesis without the complications of cyclicity and high conversion to estrogens. The Institutional Animal Care and Research Advisory Committee of the University of Texas Southwestern Medical Center approved all animal protocols. All mice used in experiments were fed a standard irradiated chow diet and housed in a temperature-controlled environment with a 6 am to 6 pm light-dark cycle. Unless specified, mice used in experiments were 6–8 weeks old.

Fertility was assayed by placing fertile WT female mice with individually housed male mice. Breeding cages were surveyed daily for the presence of pups that if found were removed and killed. Adult animals were killed by asphyxiation with isoflurane and then exsanguination via cardiac puncture; pups were killed by decapitation. For stimulation of steroidogenesis, human chorionic gonadotropin (hCG) (Sigma) was dissolved in a 0.9% saline solution for a final concentration of 100 mIU/mL. Mice were administered 100 μL of hCG solution or vehicle via an ip injection and killed 2 hours later. Tissue fixation, hematoxylin and eosin staining, and microscopy for histological analyses were conducted according to standard protocols.

### RT-PCR, immunoblotting, urine and blood collection, and dynamic testing

For RNA extraction, frozen liver or testis samples were homogenized in 500 μL of RNA-STAT60 (Isotex Diagnostics), 100 μL of chloroform was added, and the phases were separated by centrifugation. The upper phase was removed, and RNA was precipitated with 500 μL of ice-cold n-propanol. After centrifugation, the pellet was washed with 70% ethanol, followed by deoxyribonuclease treatment and reverse transcription using random hexamers to generate cDNAs. Reverse transcriptase-quantitative PCR analyses used 25 ng of cDNA and 150 nmol of primers mixed with SYBR GreenER PCR Master Mix (Invitrogen). Reactions were performed on an ABI PRISM 7900 HT (Applied Biosystems), and mRNA levels were calculated by normalization to cyclophilin using the comparative cycle threshold method. *Cyb5* primers were designed using Primer Express (Applied Biosystems): 5′-CGATCTGACCAAGTTTCTCGAA-3′ and 5′-CCCCAGCTTGCTCTCTTAGG-3′.

For protein analyses, frozen livers were homogenized in protein-lysis buffer containing 10mM Tris-HCl (pH 7.5), 150mM NaCl, 0.5% 4-nonylphenyl poly(ethylene glycol), 10% glycerol, 5mM EDTA, and 1 complete miniprotease inhibitor tablet per 10 mL (11836153001; Roche). Samples were homogenized and centrifuged at 13 000*g* for 20 minutes. The supernatant was assayed for protein concentration using a colorimetric assay. Testis homogenates used for enzyme assay (see below) were mixed 2:1 with sample buffer (Laemmli sample buffer; Bio-Rad) and boiled for 5 minutes. For immunoblots, 40–50 μg of protein were resolved on 4%–20% Tris-glycine SDS-PAGE gels and transferred to polyvinylidene difluoride membranes at 7 V for 1 hour. Incubation with primary rabbit polyclonal human anti-Cyb5 antibody (1:500; Abcam) was performed in Tris-buffered saline containing 0.05% Tween and 3% BSA. After washing, incubation with secondary goat antirabbit IgG horseradish peroxidase-conjugated antibody (1:10 000; PerkinElmer) was performed in Tris-buffered saline containing 0.1% Tween and 5% fat-free milk. Clarity Western enhanced chemiluminescent substrate (Bio-Rad) was used to visualize results. HRP-conjugated anti-β-actin antibody was used as a loading control (1:1000; Cell Signaling).

Blood obtained through cardiac puncture as previously described was collected in EDTA-coated microfuge tubes. Plasma was separated and used for steroid profiling. Urine was collected from the wells of 96-well plates placed at the base of cages with individual mice. Mice were placed in the urine-collection cages for 2 hours a day for 5 days to minimize stress, and urine collected from the same mouse on different days was pooled for analysis.

### Enzyme assay

Recombinant tetrahistidine-tagged human b5 was prepared as described (27). Testes (80–130 mg) were homogenized in 0.25 mL of 0.25M sucrose containing 10mM Tris-HCl (pH 7.0) and 1mM EDTA with 10 strokes of Teflon homogenizer. The debris was pelleted by centrifuging 3 minutes at 5000*g* and discarded, and the decanted crude homogenate was stored frozen at −20°C until use. Aliquots (10 μL) were incubated with 10μM [^3^H]-labeled Prog or 17OHP (100 000 counts per minute per incubation) and 1mM reduced nicotinamide adenine dinucleotide phosphate in 50mM potassium phosphate buffer (pH 7.4) with and without 2–30 pmol b5 (estimated 5 pmol b5/pmol P450 17A1 based on activity) at 37°C in a total volume of 0.5 mL for 30 minutes. Steroids were extracted with 1 mL 1:1 ethyl acetate/isooctane or 1 mL dichloromethane. After centrifugation at 8000 rpm for 1 minute, the organic phase was transferred into glass tubes and concentrated under nitrogen. Samples were reconstituted with 20 μL methanol, and 5-μL samples were injected into an Agilent 1260 Infinity HPLC system equipped with UV detector and β-RAM4 in-line scintillation counter (LabLogic). Steroid standards and samples were resolved on a Kinetex 50 × 2.1 mm, 2.6 μm particle size C_8_ column (Phenomenex) and methanol-water gradients as described (28), mixed with Bio-SafeII scintillation cocktail (Research Products International), and quantitated by integration of radioactivity peaks using Laura4 software (LabLogic).

### Plasma steroid profiling by liquid chromatography-tandem mass spectrometry (LC-MS/MS)

A 50-μL aliquot of plasma was deproteinated with acetonitrile and methanol containing deuterated internal standards, and steroids were extracted and quantitated using an Agilent 1290 binary pump HPLC attached to an Agilent 6490 triple quadrupole tandem mass spectrometer as described (29, 30). Assay performance characteristics and precursor/product ion pairs were reported in these references.

### Urine steroid metabolite profiling by gas chromatography-mass spectrometry

Steroids in a 0.5- to 1-mL sample of urine were extracted, derivatized, and quantitated as previously described (31). In mice, the major androgen metabolite is an incompletely characterized androstanetriolone, which when fully silylated gives a molecular ion of M^+^ = 416 m/z. Excretion of metabolites in untimed samples were normalized to volume and expressed as area under the curve per mL urine.

## Results

### Generation of Leydig cell b5 knockout (LeyKO) mice

**Figure 1.**
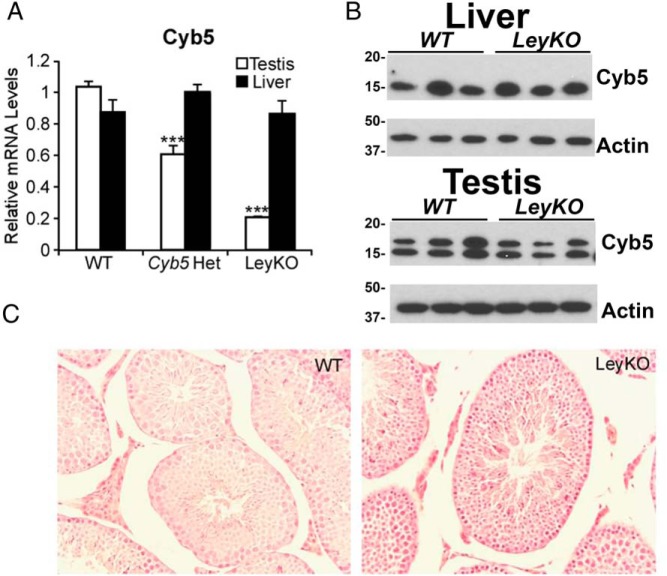
Specific deletion of b5 in testis but not liver. A, RT-qPCR analysis of *Cyb5* mRNA expression in testis and liver from mice that are *Cyb5*^flox/flox^ (WT), *Cyb5*^flox/+^:Sf1-*Cre* (*Cyb5* Het), and *Cyb5*^flox/flox^:Sf1-*Cre* (LeyKO). Data are mean ± SD, and statistics were determined with 2-tailed *t* test (***, *P* < .0005). B, Immunoblot analysis of b5 protein in liver and testis homogenates from WT and LeyKO mice, using β-actin as a loading control and migration of molecular mass standards (kDa) at left. C, Hematoxylin and eosin staining of testis sections from WT and LeyKO mice both show normal histology (magnification 20×).

To study the in vivo role of microsomal b5 in testicular steroid synthesis, we generated LeyKO mice by crossing *Cyb5*^flox/flox^ and Sf1-*Cre* transgenic strains. *Cyb5* mRNA was reduced 40% in testis from *Cyb5*^flox/+^:Sf1-*Cre* mice and 80% in *Cyb5*^flox/flox^:Sf1-*Cre* mice (subsequently referred to as LeyKO), whereas expression in the liver was unchanged (Figure 1A). Immunoreactive b5 proteins appeared similar in homogenates of liver and testis from LeyKO mice compared with floxed littermates, with 2 major b5 species in testis from all animals (Figure 1B). Because Leydig cells compose only approximately 5% of testis mass, the residual b5 expression might derive from other cell types, which comprise most testis tissue, or from incomplete recombination. LeyKO mice were viable and born at the expected Mendelian ratio. LeyKO male mice were observed to be phenotypically normal, with no differences in internal or external sexual development between knockout mice and floxed littermates.

### LeyKO mice have normal body weight, sex organ weight, and fertility

Testicular histology revealed no defects in testicular development in LeyKO mice (Figure 1C). To determine the impact on sexual maturation, we measured the weight of the epididymis, vas deferens, seminal vesicles, preputial glands and testis from LeyKO mice and compared them with floxed littermates (Table 1). No significant differences were observed between the 2 groups. Additionally, no differences in body weight were observed between LeyKO and floxed littermates (Table 1). Decreased androgen production would be expected to relieve feedback inhibition on the hypothalamus and pituitary, leading to an increase in gonadotropins; however, no increase in FSH or LH was seen in LeyKO mice (Table 1). To determine whether LeyKO mice had any defects in their ability to reproduce, we individually housed LeyKO and floxed littermates with WT females and measured litter size and frequency. No differences in litter size or frequency were observed between the 2 cohorts (Table 1), indicating that LeyKO mice had no defect in their ability to copulate. These results are consistent with previously published data on global b5-null mice that were found to be viable, fertile, and anatomically indistinguishable from WT mice (23).

**Table 1.** Characterization of LeyKO Male Mice vs Littermates

**Sex organ and body weights**				
	WT (n = 9)		LeyKO (n = 8)	
	Mean (mg)	SD	Mean (mg)	SD
Vas deferens	5.0	0.6	4.8	0.6
Epididymis	15.5	6.5	14.6	2.2
Seminal vesicle	34.6	6.7	32.9	10.5
Preputial gland	25.4	5.8	23.4	8.2
Testis	39.3	7.3	36.5	7.7
Body weight	24 600	3500	26 200	5100
**Plasma FSH/LH**				
	WT (n = 5)		LeyKO (n = 9)	
	Mean (ng/mL)	SD	Mean (ng/mL)	SD
LH	0.16	0.15	0.27	0.32
FSH	106	37	135	54
**Fertility**				
	WT (n = 5)		LeyKO (n = 5)	
	Mean	SD	Mean	SD
Days between litters	28.2	8.5	23.2	3.6
Litter size	7.3	2.0	7.4	1.2
**Basal plasma steroids**				
	WT (n = 5)		LeyKO (n = 4)	
	Mean (ng/mL)	SD	Mean (ng/mL)	SD
Progesterone	6.92	1.92	10.2	2.83
17-hydroxyprogesterone	0	0	0.45	0.90
Testosterone	0.38	0.08	0.59	0.65
Androstenedione	0.76	0.13	0.74	0.16
11-deoxycortisol	0.83	0.47	0.86	0.19
Corticosterone	65.1	18.9	63.1	25.5
11-deoxycorticosterone	1.79	0.86	1.96	0.35

### LeyKO mice show slight changes in basal plasma and urine steroid profiles

Given the strong stimulatory effect of b5 on the P450 17A1-catalyzed 17,20-lyase reaction, we anticipated that deletion of b5 from Leydig cells would decrease circulating 19-carbon steroids AD and T and lead to an accumulation of 21-carbon precursors, particularly 17OHP. Using LC-MS/MS to profile plasma steroids, we found neither a significant decrease in AD and T nor a significant increase in 17OHP and Prog (Table 1). Furthermore, plasma 17OHP concentrations were consistently low or undetectable (<0.5 ng/mL) in both LeyKO mice and floxed littermates, and in agreement with previous studies, plasma T did not exceed 0.5–1 ng/mL (32). These results suggest that residual 17,20-lyase activity in the absence of b5 was sufficient to maintain the low basal level of T synthesis in male mice without accumulation of precursors. An alternative explanation is that b5-dependent extratesticular androgen synthesis compensates for the lack of testicular AD and T production in LeyKO mice; however, this hypothesis is unlikely given the minor reductions in plasma T found in global b5-null animals (23). To confirm these findings, we performed urinary steroid metabolite profiling by gas chromatography-mass spectrometry, which was used to demonstrate impaired 17,20-lyase activity in patients with b5 deficiency (21). Urine samples from 10-week-old LeyKO mice and floxed littermates (n = 10 each group) showed slightly lower amounts of the major androstanetriolone androgen metabolite compared with floxed littermates (area under the curve per mL urine = 2 042 000 ± 927 000 vs 2 786 000 ± 865 000, respectively; *P* = .09). These data demonstrate minimal changes in basal steroid production in LeyKO mice.

### Stimulation of steroid production exposes accumulation of 17OHP and Prog in LeyKO mice

**Figure 2.**
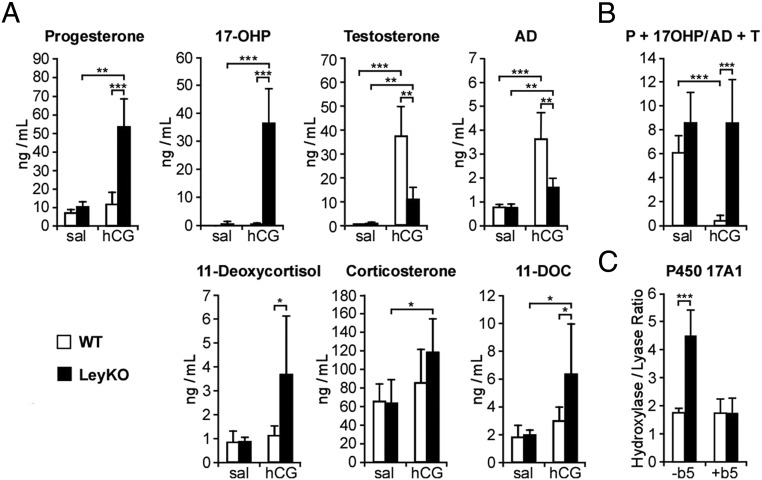
Steroidogenesis stimulation with hCG leads to accumulation of Prog and 17OHP in LeyKO mice. The *Cyb5*^flox/flox^ (WT) and LeyKO male mice 2–4 months of age (n = 5–7) were injected with 10 mIU of hCG or saline, killed 2 hours later, and exsanguinated. A, Plasma levels of Prog, 17OHP, T, AD, 11-deoxycortisol, corticosterone, and 11-deoxycorticosterone (11-DOC) determined by LC-MS/MS. B, Ratio of plasma concentrations of 21-carbon steroids (Prog + 17OHP) to 19-carbon steroids (AD + T) as a functional measure of in vivo 17,20-lyase activity. C, P450 17A1 activities, expressed as hydroxylase to lyase ratio in testicular homogenates from LeyKO and WT mice (n = 13–23). Testicular homogenates were incubated with either [^3^H]-Prog or [^3^H]-17OHP, and total products were measured by HPLC with radiochemical detection to assay 17-hydroxylase and 17,20-lyase activities, respectively (see Materials and Methods). Addition of recombinant b5 to the testicular homogenates (+b5) rescued the decreased 17,20-lyase activity in LeyKO homogenates but did not change activities in WT homogenates. Values are mean ± SD, and statistics were determined with 2-tailed *t* test (*, *P* < .05; **, *P* < .005; ***, *P* < .0005).

Unlike human beings, exposure of male mice to a receptive female or soiled bedding from a receptive female activates the vomeronasal reflex and stimulates LH release and T synthesis, with circulating T increasing to 6 ng/mL within 20–60 minutes of exposure (33, 34). Thus, basal T production rates in mice are low and a poor test for a partial blockade in 17,20-lyase activity. To simulate a transient robust burst of androgen synthesis and P450 17A1-catalyzed steroidogenesis, LeyKO mice and floxed littermates were injected with 10 mIU of hCG and killed 2 hours later, when plasma T peaks after hCG stimulation (35). Plasma T increased approximately 100-fold (to 37 ± 12 ng/mL) after hCG stimulation in floxed animals relative to saline controls, whereas AD rose only 5-fold with negligible accumulation of 21-carbon precursors 17OHP or Prog (Figure 2A). In contrast, plasma T rose only 18-fold (to 11 ± 5 ng/mL) and AD 2-fold after hCG stimulation in LeyKO mice; however, a marked accumulation of plasma 17OHP (to 36 ± 12 ng/mL) and lesser accumulation of Prog was observed, relative to a slight increase in floxed controls. Thus, hCG stimulation elicited a similar augmentation of steroidogenesis in WT and LeyKO animals, but the lack of b5 in Leydig cells of LeyKO animals impaired 17,20-lyase activity sufficiently to detain the flux of steroids above the partial block and to afford marked 17OHP accumulation. Plasma dehydroepiandrosterone was less than 0.02 ng/mL in all 23 basal or hCG-stimulated samples tested from LeyKO mice and floxed littermates (data not shown).

When analyzed as the sum of steroids before and after the 17,20-lyase reaction, the ratio of 21-carbon precursors Prog and 17OHP to the 19-carbon products AD and T ([Prog + 17OHP]/[AD+T]) was not significantly different between unstimulated LeyKO mice and floxed littermates (Figure 2B). Upon hCG stimulation of steroidogenesis, however, brisk production of the androgens AD and T is observed, with a significant decrease in the [Prog + 17OHP] to [AD+T] ratio from 6 ± 1.4 to 0.38 ± 0.46 in normal animals (Figure 2B). In contrast, hCG stimulation of LeyKO mice did not lower the elevated [Prog + 17OHP] to [AD+T] ratio relative to saline-injected controls (both >8) due to proportionate accumulation of Prog and 17OHP along with AD and T.

### Addition of b5 to testicular homogenates from LeyKO mice restores deficient 17,20-lyase activity

To confirm that loss of b5 is the Leydig cells is responsible for low 17,20-lyase activity in LeyKO animals, we assayed 17-hydroxylase and 17,20-lyase activities in testicular homogenates and expressed the results as the hydroxylase to lyase ratio for each testis sample, to correct for variations in P450 17A1 content and homogenate quality. The hydroxylase to lyase ratio increased from 1.7 in WT mice to 4.7 in LeyKO mice (Figure 2C). Addition of exogenous b5 to the testicular homogenates restored a normal hydroxylase to lyase ratio to LeyKO mice homogenates but did not change the ratio for the WT homogenates, confirming that lack of Leydig cell b5 was responsible for the observed decrease in 17,20-lyase activity.

### Fertility and hCG stimulation of aged LeyKO mice

Healthy mice have no observed decrease in plasma T with aging (36). Given their impaired 17,20-lyase activity, we hypothesized that absence of b5 might lead to decreased plasma T and consequently decreased fertility with increasing age in LeyKO mice. To examine this possibility, LeyKO mice and floxed littermates with an average age of 596 ± 25 days were subjected to the same battery of testing as described above for the younger animals. We found no differences in fertility (litter size and frequency) (data not shown), body weight, or lean weight and fat content assessed by magnetic resonance imaging between the 2 cohorts (Table 2). Similar to young adults, aged LeyKO mice had hCG-stimulated plasma T concentrations approximately 50% that of floxed littermates with an accumulation of plasma 17OHP 50-fold higher than floxed littermates (Table 2). Similarly, homogenates of aged LeyKO mouse testis had an increased hydroxylase to lyase ratio compared with floxed littermates, which was restored to normal upon addition of exogenous b5 (data not shown). Consequently, we found no significant decline in testicular function or premature failure in aged LeyKO mice.

**Table 2.** Characterization of LeyKO Aged Male Mice vs Littermates

**Body weight, fat content, lean weight, testicular weight**					
	WT (n = 5)		LeyKO (n = 5)		
	Mean (mg)	SD	Mean (mg)	SD	
Testis	77.4	17.1	84.0	10.7	
Fat content	16 259	4139	20 248	2421	
Lean weight	26 108	2367	28 752	3207	
Body weight	45 320	6391	52 340	4224	
**hCG-stimulated plasma steroids**					
	WT (n = 5)		LeyKO (n = 5)		
	Mean (ng/mL)	SD	Mean (ng/mL)	SD	*P* value
Progesterone	7.35	2.61	25.6	14.9	.027
17-hydroxyprogesterone	0.12	0.26	6.38	4.30	.012
Testosterone	10.7	8.20	5.97	2.96	.261
Androstenedione	0.23	0.13	0.06	0.06	.023
11-deoxycortisol	0.19	0.29	4.05	2.79	.015
Corticosterone	69.2	31.1	176.2	86.5	.032
11-deoxycorticosterone	1.86	1.17	13.3	9.60	.023

### Activation of alternate pathway of steroidogenesis is not responsible for normal male phenotype of LeyKO mice

An alternate pathway to dihydrotestosterone (DHT), originally described in the tammar wallaby, is also found in both rodents and human steroidogenic cells (37). The 5α-reductase isoform Srd5a1, known to convert T to DHT, also uses Prog and 17OHP as substrates to form 5α-reduced pregnanes, which are subsequently converted through a series of steps, including the P450 17A1-catalyzed 17,20-lyase reaction, into DHT. The 17,20-lyase reaction with key intermediate substrate 5α-pregnan-3α,17α-diol-20-one, however, is minimally dependent upon b5 (17). Embryonic activation of the alternate pathway to DHT in LeyKO mice might compensate for low T production, explaining the complete virilization and lack of phenotype observed in the LeyKO mice. To test this hypothesis, LeyKO mice were crossed with Srd5a1-null mice to generate *Cyb5*^flox/flox^:Sf1-*Cre*:*Srd5a1*^−/−^ mice (referred to as LeyKO/Srd5a1^−/−^). The LeyKO/Srd5a1^−/−^ mice were born in normal Mendelian ratios and had no overt differences in internal and external sexual development. Fertility experiments revealed no differences in litter frequency or size between the LeyKO/Srd5a1^−/−^ mice and *Cyb5*^flox/flox^ littermates. These results suggest that the normal sexual development in LeyKO mice is likely due to adequate fetal androgen production by residual 17,20-lyase activity despite the absence of b5 and not due to activation of the alternate pathway to DHT that bypasses the need for b5.

## Discussion

Abundant biochemical and limited genetic data have shown that the small hemo-protein b5 provides functions as a cofactor in methemoglobin reduction, fatty acid desaturation, and several cytochrome P450-catalyzed reactions in the metabolism of xenobiotics and steroid hormones (8, 10, 22, 23, 38). For the P450 17A1, b5 significantly increases the rate of the 17,20-lyase reaction that converts 21-carbon pregnanes into 19-carbon androgens. This report describes a mouse model of conditional b5 knockout in the testicular Leydig cell (LeyKO). Although LeyKO mice were found to be phenotypically identical to floxed littermates with similar basal plasma steroids, acute stimulation of steroidogenesis led to profound differences in plasma steroids using LC-MS/MS assays, with accumulation of 21-carbon pregnanes Prog and 17OHP behind the disrupted 17,20-lyase reaction and impaired androgen synthesis. In vitro studies with testicular homogenates confirmed that the biochemical phenotype resulted from the loss of Leydig cell b5. This study provides the most complete characterization of the physiologic function of b5 in activating the 17,20-lyase activity of P450 17A1.

One limitation to our study is the use of Sf1-*Cre* transgene for conditional deletion of the *Cyb5a* gene. Sf1 is also expressed in the adrenal cortex, gonadotropes, and certain regions of the hypothalamus. In mice, P450 17A1 expression in the adrenal cortex is low, as evidenced by the high plasma corticosterone in all animals (Figure 2). Although our data hint that other 21-carbon steroids of adrenal origin also accumulate in LeyKO animals after hCG stimulation, the effect is small compared with the massive rise in 17OHP. Thus, the simultaneous loss of b5 in the adrenal cortex is unlikely to confound our conclusions, whereas the preservation of hepatic b5 expression is a unique advantage of our mouse model (Figure 1). Furthermore, the hypothalamus and pituitary are not important sites of P450 activity or androgen biosynthesis from 21-carbon steroids. The poor 17,20-lyase activity in homogenates of testes from LeyKO animals and its rescue upon addition of b5 is strong evidence that loss of b5 in the Leydig cells is sufficient to explain the consistent and marked accumulation of 21-carbon steroid precursors after hCG stimulation in these mice. The residual *Cyb5a* mRNA and b5 immunoreactive protein in LeyKO testis (Figure 1, A and B) might derive from the seminiferous tubules, which compose most the testicular tissue mass. Alternatively, incomplete recombination might account for some of the residual 17,20-lyase activity in the LeyKO mice. Even if the LeyKO Leydig cells contain some b5 protein, the reduced 17,20-lyase activity in testis homogenates and its restoration upon addition of recombinant b5 support our conclusions, although our data might underestimate the true magnitude of the b5 effect. We studied only male animals due to the additional complexities introduced from the estrus cycle and dominant conversion of androgens to estrogens in the ovary of female mice. Nevertheless, subsequent experiments with female “LeyKO” animals, engineered to lack b5 in the theca and granulosa cells, would be a logical topic of future studies.

Previous work on b5 physiology has focused on its in vitro modulation of various P450s or its in vivo effects on metabolism of various xenobiotics (22). A global b5-null mouse has been described with substantial defects in hepatic and extrahepatic drug metabolism (23). The global b5-null mice were born in normal Mendelian ratios and were unexpectedly found to be fertile with no overt phenotype. Similar to observations with the global b5-null animals, LeyKO mice had normal fertility without gross or histological changes in the testis, epididymis, vas deferens, seminal vesicles, or preputial glands. Our data demonstrate that normal androgen physiology is preserved in this strain of male mice whether b5 is deleted from the Leydig cells alone or simultaneous deletion from the liver as well, which is likely to reduce T catabolism.

In contrast to these findings in mice, human patients with *CYB5A* mutations and a 46XY karyotype are born with ambiguous genitalia and are universally infertile (21, 39). The 17,20-lyase activity of microsomes isolated from LeyKO mice is significantly reduced and comparable with the decrease in 17,20-lyase activity noted in transfected HEK293 cells expressing human P450 17A1 and b5 mutation H44L (21), demonstrating that b5 is required for maximal 17,20-lyase activity in the Leydig cells of both mice and humans. The differences in sexual development and maturation observed between LeyKO mice and human patients potentially stem from differences in the specific androgen concentrations required for fetal development. Decades of research has revealed that human fetal male sexual development is dependent on the production of T and its subsequent conversion to DHT via steroid 5α-reductase type 2 (40). Human amniotic T concentrations, measured between 11 and 21 weeks gestation, average 0.17 ng/mL for male fetuses and 0.1 ng/mL for female fetuses (41). Direct measurements of fetal T from fetal vein sampling between 15 and 38 weeks of gestation average 0.61 ng/mL for male fetuses and 0.22 ng/mL for female fetuses (42). T concentrations below a threshold lead to graded degrees of impaired virilization, which are inversely correlated. Male mouse fetal testes predominately produce T plus 5α-androstene-3α,17β-diol at day 16 and 17, when virilization of the mouse urogenital tract is known to occur (43); however, targeted disruption of both Srd5a1 and Srd5a2 does not impair virilization (44). Furthermore, one-third of mice engineered to express a phosphorylation-deficient steroidogenic acute regulatory protein are normally virilized, despite undetectable postnatal T synthesis (45). Thus, mouse virilization is exquisitely sensitive to T and does not require DHT as in humans. Consequently, the reduced T production in LeyKO animals is still sufficient to allow normal virilization of male mice.

In summary, we have shown impaired in vivo and in vitro 17,20-lyase activity in testes of LeyKO male mice, with marked accumulation of 21-carbon steroids after hCG stimulation. Despite a 200-fold increase in the stimulated [Prog+17OHP] to [AD+T] (precursor to product) ratio compared with WT littermates, the lack of b5 in Leydig cells does not affect sexual maturation, fertility, or basal steroid levels of male mice. In contrast to the impaired virilization and fertility of patients with b5 deficiency, the residual 17,20-lyase activity of the LeyKO mice is sufficient to maintain normal androgen physiology in male animals. We conclude that LeyKO mice represent a good model for the biochemistry but not the physiology of b5 deficiency in human beings, which is one of the rarest disorders of steroidogenesis.
